# Integrated Chinese and Western Medicine for Breast Cancer Patients with Depression—Association with Survival and Healthcare Utilization: A Nationwide Retrospective Cohort Study in Taiwan

**DOI:** 10.3390/healthcare14101406

**Published:** 2026-05-20

**Authors:** Chingying Liang, Yen-Chun Huang, Jiun-Liang Chen, Chi Wen Chen, Mingchih Chen

**Affiliations:** 1 Graduate Institute of Business Administration, College of Management, Fu Jen Catholic University, New Taipei City 242062, Taiwan; evonny_65@yahoo.com.tw; 2 Tenmot Tang Chinese Medicine Clinic, New Taipei City 22029, Taiwan; 3 Department of Artificial Intelligence, Tamkang University, New Taipei City 251301, Taiwan; hivicky92@gmail.com; 4 Department of Traditional Chinese Medicine, Chang Gung Memorial Hospital, Chang Gung University, Taoyuan City 33302, Taiwan; a12015@cgmh.org.tw; 5 Department of Physical Education, Fu Jen Catholic University, New Taipei City 242062, Taiwan; 055341@mail.fju.edu.tw; 6 Artificial Intelligence Development Center, Fu Jen Catholic University, New Taipei City 242062, Taiwan

**Keywords:** breast cancer, depression, integrated Chinese and Western medicine, traditional Chinese medicine, overall survival, healthcare utilization, medical expenditure

## Abstract

**Background**: Breast cancer (BC) survivors frequently experience depression, which is associated with poorer quality of life (QoL), increased healthcare utilization, and worse prognosis. Although traditional Chinese medicine (TCM) is commonly used as an adjunctive therapy among Chinese populations for cancer-related symptom relief and supportive care, population-based evidence remains limited regarding whether integrated Chinese and Western medicine (ICWM) confers measurable benefits over Western medicine (WM) alone in terms of healthcare utilization and survival. Taiwan’s National Health Insurance (NHI) system offers a unique nationwide setting to address this gap because it reimburses patients for both WM and TCM services and captures care from a large number of TCM clinics across Taiwan, allowing evaluation of adjunctive TCM use in routine clinical practice at a scale rarely possible in prior studies. We used emergency department visits, hospitalization, and length of stay as pragmatic proxy indicators of patients’ daily functioning and disease burden. Leveraging a 10-year enrollment window (2004–2013) and up to 17 years of follow-up, we hypothesized that ICWM would be associated with a reduced risk of acute care events and lower healthcare expenditures compared with WM alone. This hypothesis was examined in a large cohort of breast cancer patients treated across nearly 4000 medical facilities nationwide, encompassing the entire Taiwanese population. **Methods**: A retrospective cohort study was performed to analyze Taiwan’s National Health Insurance Research Database and Cancer Registry. Women newly diagnosed with breast cancer between 2004 and 2013 who subsequently developed depression (≥3 outpatient diagnoses or 1 hospitalization) were followed until death or 31 December 2021. Patients receiving ≥30 cumulative days of TCM after diagnosis were classified as the ICWM group, whereas those receiving <30 days were classified as the WM group. Multivariable Cox proportional hazards models were used to estimate adjusted hazard ratios (aHRs) for all-cause mortality. Healthcare utilization, including emergency department visits, hospitalization, and medical expenditures, was analyzed on a per-person-year basis. **Results**: A total of 1193 patients were included, with 488 in the WM group and 705 in the ICWM group. Compared with WM users, ICWM users were younger, had lower body mass index, and were more likely to have stage 0–II disease. ICWM was associated with lower total, inpatient, and emergency healthcare expenditures per person-year, as well as fewer emergency visits per person-year, although outpatient and overall visits were higher. In stage-stratified multivariable analyses, ICWM was associated with lower all-cause mortality in both stage 0–II disease (aHR = 0.61, 95% CI: 0.39–0.94) and stage III–IV disease (aHR = 0.38, 95% CI: 0.21–0.67). Kaplan–Meier analyses likewise showed significantly better overall survival in the ICWM group in both early-stage and advanced-stage disease. **Conclusions**: In this nationwide retrospective cohort of breast cancer patients with depression, adjunctive ICWM was associated with better survival, lower acute care utilization, and lower healthcare expenditures compared with WM alone. However, because quality of life was not directly measured and the study was based on observational data, QoL-related interpretations should be made cautiously, with healthcare utilization outcomes viewed as indirect proxy indicators rather than direct evidence of improved daily QoL.

## 1. Introduction

Breast cancer (BC) remains one of the most prevalent malignant tumors among women and continues to impose a substantial global health burden [1,2,3,4]. In 2022, approximately 2.26 million new cases were diagnosed worldwide, with an estimated 665,684 deaths, accounting for about 6.9% of all cancer-related mortality [2,5]. According to recent global cancer statistics, BC has surpassed lung cancer to become the most frequently diagnosed cancer worldwide for the first time [2,5].

In WM, current treatment strategies typically include surgery, chemotherapy, radiotherapy, and hormone therapy, administered alone or in combination based on tumor characteristics and disease stage [6]. Mastectomy remains a cornerstone of breast cancer management for many patients; however, it may affect body image and remove traditional symbols of femininity, thereby diminishing self-esteem and evoking feelings of shame and vulnerability [7,8,9]. These psychosocial consequences contribute to substantial psychological distress across the illness trajectory [9,10,11]. Depression is one of the most common comorbidities among patients with BC and has been associated with poorer psychological well-being, greater healthcare utilization, and reduced quality-of-life (QoL) [12,13]. In recent years, integrated Chinese and Western medicine (ICWM) has been increasingly implemented in clinical settings, representing a therapeutic strategy that synergistically combines Western medicine (WM) with traditional Chinese medicine (TCM) [14]. Across most Asian countries, TCM serves as the predominant form of complementary and integrative therapy [15].

Studies indicate that patients with breast cancer have a significantly higher incidence of depression than those with colorectal, prostate, or other malignancies, with global prevalence estimates of 30–32% and an overall prevalence of long-term depression following treatment of 39.9% [16,17,18]. During therapy, the prevalence of depression among breast cancer patients may increase to as high as 60%, and cancer patients with depression tend to have higher overall medical expenditures [19,20,21,22]. Depression is a common comorbidity among Chinese populations in patients with cancer, and TCM is frequently used as an adjunctive modality for symptom relief and supportive care [23,24]. Although TCM is widely used as an adjunctive therapy in Chinese societies, few studies have examined whether such adjunctive treatment may significantly alleviate depression-related burden in patients with BC. In particular, incident depression after breast cancer diagnosis may more directly reflect the psychological and clinical burden arising from the cancer trajectory, including diagnostic stress, treatment-related adverse effects, changes in daily functioning, and subsequent emotional distress [11,12]. Focusing on patients with new-onset post-diagnosis depression may accordingly provide a more clinically relevant framework for evaluating the role of integrative care in this vulnerable subgroup.

Despite growing recognition of the mental health burden among breast cancer survivors, studies rigorously evaluating both treatment outcomes and healthcare expenditures of ICWM compared with WM alone in patients with breast cancer and comorbid depression remain scarce. This gap is important because depression in patients with breast cancer is associated not only with substantial psychological distress but also with increased healthcare utilization and medical costs, making expenditure assessment highly relevant from both clinical and health system perspectives.

Taiwan’s National Health Insurance (NHI) system, established in 1995, currently covers more than 99.6% of the population and reimburses both WM and TCM services, thereby providing a highly comprehensive, population-based healthcare claims database [23,25]. This nationwide integration provides an important opportunity to examine the real-world association of ICWM with both clinical and economic outcomes.

In addition to survival and other clinical endpoints, healthcare expenditures are a particularly meaningful outcome because they reflect the cumulative burden of treatment, complications, emergency care, hospitalization, and long-term resource use at both the patient and system levels [26]. In this study, real-world healthcare utilization indicators—such as emergency department (ED) visits, hospitalization, and length of hospital stay—were used as pragmatic proxy measures of patients’ disease burden, functional status, and health-related outcomes, rather than as direct measurements of patient-reported quality of life.

Furthermore, in Taiwan, TCM is predominantly delivered in clinic-level settings rather than hospital-based departments, and the National Health Insurance Research Database (NHIRD) captures claims from nearly all contracted healthcare institutions, including a large number of TCM clinics [23,25]. This comprehensive coverage provides a rare opportunity to evaluate adjunctive TCM use across diverse real-world practice settings. In addition, the 10-year enrollment window and follow-up of up to 17 years allowed a more comprehensive assessment of long-term clinical outcomes, healthcare expenditures, and the cumulative impact of depression and integrative treatment on healthcare use and QoL-related trajectories than is typically feasible in smaller, shorter-term investigations.

Therefore, we aimed to compare all-cause mortality and healthcare expenditures between ICWM and WM alone among patients with breast cancer and comorbid depression using Taiwan’s nationwide NHI claims data. Given that real-world healthcare utilization may partially reflect patients’ functional status, symptom burden, and everyday well-being, we further examined whether adjunctive ICWM use was associated with reduced emergency department utilization, fewer or shorter hospitalizations, and lower overall medical expenditures. These outcomes were interpreted as pragmatic indicators of healthcare burden and utilization patterns, rather than direct measures of QoL. Specifically, we aimed to (1) estimate and compare key clinical outcomes between ICWM and WM; (2) quantify and compare total healthcare expenditures and major cost components between groups; and (3) identify patient and treatment characteristics associated with the use of ICWM and with subsequent outcomes and expenditures.

## 2. Materials and Methods

### 2.1. Data Sources

This study used a retrospective cohort design based on Taiwan’s National Health Insurance Research Database (NHIRD), a de-identified, population-based claims database that has covered more than 99.9% of Taiwan’s 23 million residents since 1995. Administered by the National Health Research Institutes (Taipei, Taiwan), the NHIRD contains longitudinal data on demographics, outpatient and inpatient claims, diagnoses, procedures, and prescriptions, enabling robust population-based epidemiologic research. The Longitudinal Health Insurance Research Database (LHIRD), a subset of the NHIRD, comprises data from two million beneficiaries randomly sampled from the 2000–2021 registry of all National Health Insurance enrollees, with stratified random sampling by age and sex to ensure representativeness of the full NHIRD population [27]. Diagnoses were coded using ICD-9-CM through 2015 and ICD-10-CM thereafter [25,28].

To identify breast cancer cases, the NHIRD was further linked to the Taiwan Cancer Registry (TCR) [25], a nationwide population-based cancer registry established in 1979 by the Ministry of Health and Welfare (Taipei, Taiwan) that collects comprehensive information on cancer incidence, treatment, and survival across Taiwan [29]. The registry serves as a key data source for cancer epidemiology and outcome research. Cancer sites were classified according to the International Classification of Diseases for Oncology (ICD-O). The TCR demonstrates high data quality, with a coverage rate of 98.4%, and all cancer sites have a rate of 93.0% and 97.6%, respectively, reflecting excellent data completeness and reliability [29,30].

### 2.2. Ethics Statement

All relevant claims pertaining to beneficiary data underwent rigorous encryption and irreversible anonymization prior to analysis, ensuring full compliance with data privacy requirements and minimizing any risk of participant re-identification. Accordingly, the Institutional Review Board of Shin-Kong Wu Ho-Su Memorial Hospital granted a waiver of informed consent for this minimal risk, secondary data research (IRB approval number: 20240702R), in accordance with current regulatory and ethical standards.

### 2.3. Study Design and Population

In this study, patients with breast cancer were first identified from the Taiwan Cancer Registry (TCR) using ICD-9-CM codes 174 and 175 or ICD-O code C50. These records were then linked to the ambulatory and inpatient claims files of the LHIRD to identify underlying comorbidities, as well as healthcare utilization. Then, mortality status was ascertained through linkage with the cause of death data.

**Figure 1:**
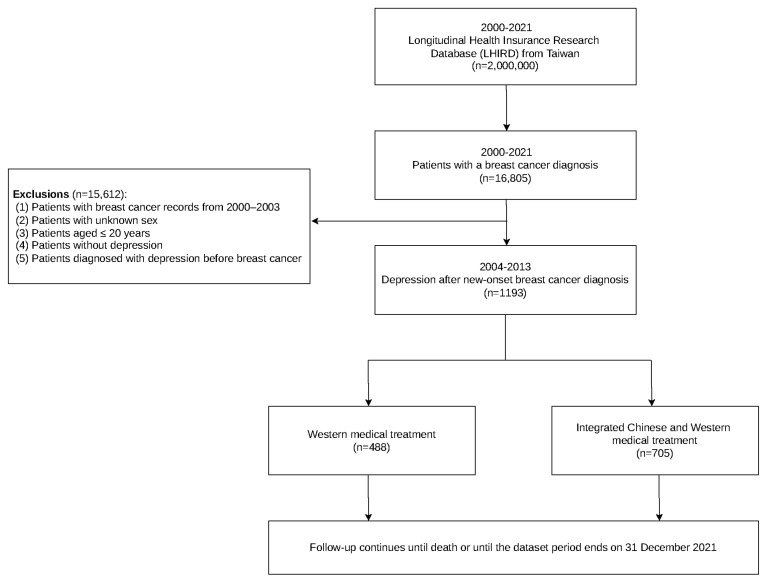
Flowchart of the study population selection process for breast cancer patients with new-onset depression receiving different treatment modalities.

As shown in Figure 1, 16,805 patients diagnosed with breast cancer were initially identified from the cohort. The exclusion criteria were as follows: patients with BC records dated 2000–2003; unknown sex; age ≤20 years; no depression; or depression diagnosed before the breast cancer diagnosis. After applying these criteria, 1193 patients who developed depression following a new diagnosis of breast cancer between 2004 and 2013 were eligible for inclusion. Depression was identified using ICD-9-CM or ICD-10-CM diagnostic codes, with case ascertainment requiring at least three outpatient diagnoses or one inpatient diagnosis to improve diagnostic specificity and reduce the likelihood of miscoding from a single claim [31]. Thus, the study cohort was restricted to breast cancer patients who subsequently developed depression. Eligible patients were then categorized according to Chinese herbal medicine use after breast cancer diagnosis. Adjuvant Chinese herbal medicine treatment was defined as the use of Chinese herbal medicine for at least 30 cumulative days after breast cancer diagnosis. Patients with fewer than 30 cumulative days of use were classified into the WM group, whereas those with 30 cumulative days or more were classified into the ICWM group [32,33]. Of the included patients, 488 received WM only, whereas 705 received both TCM and WM. All participants were followed from the breast cancer index date until death or 31 December 2021.

### 2.4. Definition of Medical Expenses

To minimize bias arising from expenditure variability during active treatment and to better capture the late-stage healthcare burden, medical expenses were defined as all healthcare expenditures incurred from the day after the final treatment through death or the end of follow-up, excluding the final treatment itself. This operational definition enables a more precise assessment of how advanced disease and comorbid depression influence healthcare resource utilization.

For each individual, total spending was aggregated over the observed follow-up period and standardized by person-time to account for differences in follow-up duration, yielding average annual expenditures (per person-year). Analyses were conducted for both total medical expenditures and category-specific outlays (outpatient, inpatient, and emergency) to characterize utilization patterns and expenditure disparities among breast cancer patients with comorbid depression.

### 2.5. Definitions of Baseline Variables

Baseline variables included follow-up time, age group (<50, 50–64, and ≥65 years), age, height, weight, body mass index (BMI), Charlson Comorbidity Index (CCI) score, treatment modality, cancer stage, insured category, taxane exposure, and underlying diseases. BMI was calculated as weight in kilograms divided by height in meters squared and was analyzed both as a continuous variable and as a categorical variable. BMI categories were defined as underweight (<18.5 kg/m^2^), normal weight (18.5–24.9 kg/m^2^), overweight (≥25.0 kg/m^2^), and unknown. CCI scores were calculated from claims-based diagnosis codes using a Charlson Comorbidity Index algorithm compatible with ICD-9-CM and ICD-10-CM coding and were categorized as 0–1, 2–3, 4–5, and ≥6. Cancer stage was obtained from the Taiwan Cancer Registry and grouped as stage 0–II, stage III–IV, and unknown. Treatment modality was classified using a surgery-based framework because surgery is the principal treatment for most nonmetastatic breast cancers, with additional therapies administered as adjuvant treatment in selected patients. Accordingly, treatment modality was categorized as operation only (OP only), operation plus radiotherapy (OP + RT), operation plus hormone therapy (OP + HT), operation plus chemotherapy (OP + CT), and others. The category “others” included treatment combinations that were not classified separately because cell sizes in more detailed categories were too small for reporting under data release regulations. Taxane exposure was defined as receipt of taxane-based chemotherapy according to treatment claims records. Insured category was classified as employer/professional, general, and parental leave/dependent according to insurance registry records. Underlying diseases were identified from ambulatory or inpatient claims and included hypertension, hyperlipidemia, congestive heart failure, diabetes mellitus, gout, pneumonia, major bleeding, gastrointestinal bleeding, anemia, and ischemic heart disease, the detailed ICD-9 and ICD-10 codes are listed in Appendix A.

### 2.6. Statistical Analysis

Categorical variables were expressed as counts and percentages and compared using the Chi-square test. Continuous variables were presented as mean ± standard deviation (SD), and skewed variables were additionally summarized as median and interquartile range (IQR). Because the primary outcome of interest was mean medical expenditure, between-group differences were assessed using the independent-samples Student’s *t*-test. Survival probabilities were estimated using the Kaplan–Meier method, and between-group differences were assessed using the log-rank test. The Kaplan–Meier analyses were conducted using the same study cohort and the same time origin as the Cox proportional hazards models, with survival time defined from the breast cancer index date to all-cause mortality or the end of follow-up. In the Cox proportional hazards regression models, time to event was defined as the interval from the breast cancer index date to all-cause mortality, with patients who remained alive at the end of follow-up treated as censored observations. Adjusted hazard ratios (aHRs) and 95% confidence intervals (CIs) were calculated accordingly. All statistical analyses were performed using SAS software version 9.4 (SAS Institute, Cary, NC, USA), and a two-sided *p*-value <0.05 was considered statistically significant.

## 3. Results

### Study Population Characteristics

Based on the analysis of 1193 breast cancer patients with subsequent depression, significant differences were observed between patients receiving WM alone (n = 488) and those receiving combined TCM and WM (n = 705) in several baseline and clinical characteristics. The ICWM group was significantly younger (mean age: 51.31 vs. 54.95 years, *p* = 0.001) and contained a higher proportion of patients aged below 50 years (63.55% vs. 52.05%, *p* = 0.001). Body mass index (BMI) was lower in the ICWM group (23.76 vs. 24.68, *p* = 0.001). Disease stage distribution differed significantly between groups (*p* = 0.021); 80.0% of the ICWM group and 76.23% of the WM group were diagnosed with stage I–II disease, while advanced stages (III–IV) were more frequent in the WM group (19.88% vs. 14.33%). Furthermore, initial treatment modalities varied significantly (*p* = 0.006). Analysis of underlying diseases revealed significant disparities across multiple conditions. The WM group exhibited a higher prevalence of hypertension (11.48% vs. 5.67%, *p* < 0.001), hyperlipidemia (36.48% vs. 30.35%, *p* = 0.027), congestive heart failure (CHF) (6.35% vs. 3.40%, *p* = 0.017), diabetes mellitus (25.41% vs. 15.32%, *p* = 0.001), gout (17.01% vs. 11.77%, *p* = 0.010), and ischemic heart disease (IHD) (20.08% vs. 15.18%, *p* = 0.027). Overall, the WM and ICWM groups differed in several baseline demographic and clinical characteristics as shown in Table 1.

Table 2 summarizes healthcare expenditures in the WM and ICWM groups. Patients in the ICWM group had a longer overall follow-up time than those in the WM group (10.61 ± 2.46 vs. 9.99 ± 3.16 years). The mean total expenditure per person-year was lower in the ICWM group than in the WM group (114,145 ± 189,454 vs. 162,084 ± 268,369), whereas the median expenditure per person-year was similar between groups (48,208 [IQR: 25,774–90,174] vs. 50,513 [IQR: 24,950–117,302]).

Regarding inpatient care, the ICWM group also had a longer follow-up time (10.46 ± 2.60 vs. 9.88 ± 3.39 years) and lower expenditure per person-year than the WM group, based on both the mean (58,381 ± 125,912 vs. 96,916 ± 208,929) and median (7165 [IQR: 0–24,640] vs. 10,347 [IQR: 0–40,674]). Emergency care showed a similar pattern, with lower mean and median expenditure per person-year in the ICWM group (1588 ± 2688 and 296.7 [IQR: 0–1163], respectively) than in the WM group (2752 ± 5790 and 467.4 [IQR: 0–1937], respectively).

By contrast, outpatient expenditure per person-year was more comparable between groups. The mean outpatient expenditure per person-year remained lower in the ICWM group than in the WM group (74,566 ± 123,256 vs. 89,397 ± 141,724), whereas the median outpatient expenditure per person-year was slightly higher in the ICWM group (41,345 [IQR: 26,850–67,107] vs. 39,884 [IQR: 24,132–69,612]). Overall, these descriptive results suggest lower total healthcare expenditures in the ICWM group, with the difference appearing to be driven mainly by lower inpatient and emergency care expenditures.

As shown in Table 3, the total number of healthcare visits was 142,382 in the WM group and 253,291 in the ICWM group. The mean number of overall visits per person was higher in the ICWM group than in the WM group (39.63 ± 20.00 vs. 34.14 ± 18.78). The mean number of inpatient visits per person was lower in the ICWM group than in the WM group (1.44 ± 2.69 vs. 1.94 ± 4.41), and the median values were similar (0.5 [IQR: 0.2–1.4] vs. 0.6 [IQR: 0.3–1.5]). The ICWM group had a higher mean number of outpatient visits per person than the WM group (38.62 ± 19.34 vs. 32.64 ± 17.24), with corresponding median values of 34.3 [IQR: 24.7–48.1] and 29.2 [IQR: 21.2–39.8]. Emergency visits showed the opposite pattern, with a lower mean number of visits per person in the ICWM group than in the WM group (0.43 ± 0.50 vs. 0.63 ± 1.09). The median number of emergency visits per person was also lower in the ICWM group (0.3 [IQR: 0.1–0.5] vs. 0.4 [IQR: 0.2–0.6]).

Table 4 presents the results of the stage-stratified multivariable Cox proportional hazards regression analyses for all-cause mortality. All listed covariates were entered simultaneously into each model, and hazard ratios were estimated relative to the corresponding reference categories. In the stage 0–II subgroup, patients receiving ICWM exhibited a significantly lower risk of all-cause mortality compared with those receiving WM (adjusted hazard ratio [aHR] = 0.61, 95% confidence interval [CI]: 0.39–0.94, *p* = 0.0242). Older age (≥65 years) was strongly associated with an increased mortality risk (aHR = 3.10, 95% CI: 1.72–5.59, *p* < 0.001) compared with patients aged <55 years. Among comorbidities, CHF remained a significant predictor of mortality (aHR = 2.95, 95% CI: 1.53–5.72, *p* = 0.0013). Other comorbidities, including hypertension, diabetes, and major bleeding, were not significantly associated with the outcome in this subgroup. In the Stage III–IV subgroup, the protective effect of ICWM persisted and appeared even more pronounced (aHR = 0.38, 95% CI: 0.21–0.67, *p* < 0.001) compared with WM. In this advanced-stage cohort, age was not a statistically significant factor (≥65 years: aHR = 2.04, *p* = 0.0887). Interestingly, patients with diabetes showed a significantly lower all-cause mortality risk in this subgroup (aHR = 0.29, 95% CI: 0.13–0.68, *p* = 0.0042).

**Figure 2:**
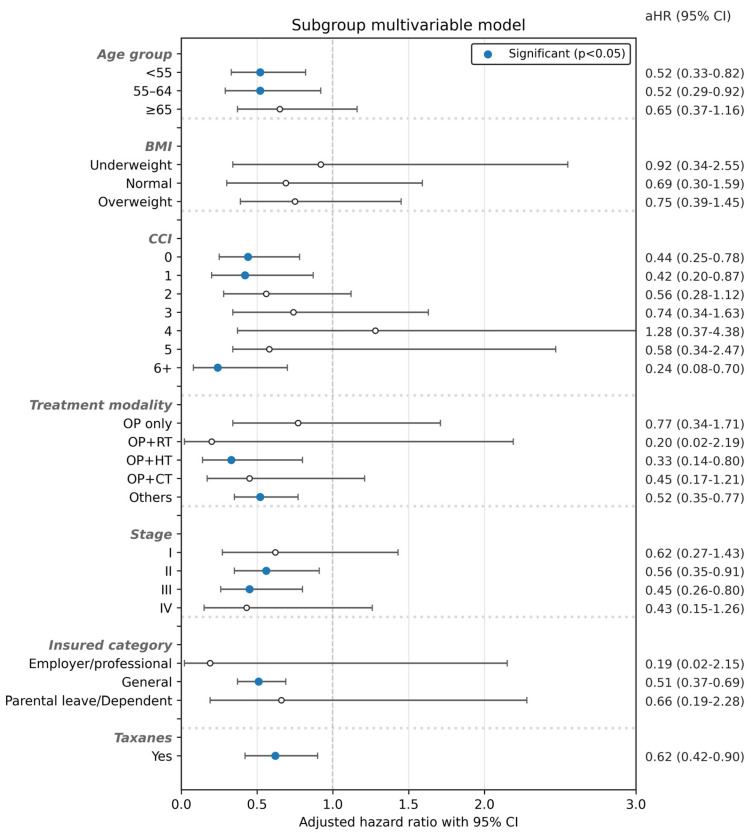
Subgroup analysis of WM and ICWM treatments and mortality risk in BC patients with depression.

In the subgroup analysis shown in Figure 2, we examined the association between treatment modality (WM vs. ICWM) and mortality risk among patients with breast cancer and depression. In age-stratified analyses, ICWM was associated with a significantly lower mortality risk among patients aged <55 years (adjusted hazard ratio [aHR] = 0.52, 95% confidence interval [CI]: 0.33–0.82; *p* = 0.004) and 55–64 years (aHR = 0.52, 95% CI: 0.29–0.92; *p* = 0.024). Among treatment modality subgroups, a significant association was observed in the OP + HT subgroup (aHR = 0.33, 95% CI: 0.14–0.80; *p* = 0.014). Stage-specific associations were also observed among patients with stage II disease (aHR = 0.56, 95% CI: 0.35–0.91; *p* = 0.020) and stage III disease (aHR = 0.45, 95% CI: 0.26–0.80; *p* = 0.006), as well as among taxane recipients (aHR = 0.62, 95% CI: 0.42–0.90; *p* = 0.011). However, not all subgroup estimates reached statistical significance, and these findings should be interpreted as exploratory, the detailed table are listed in Appendix B.

**Figure 3:**
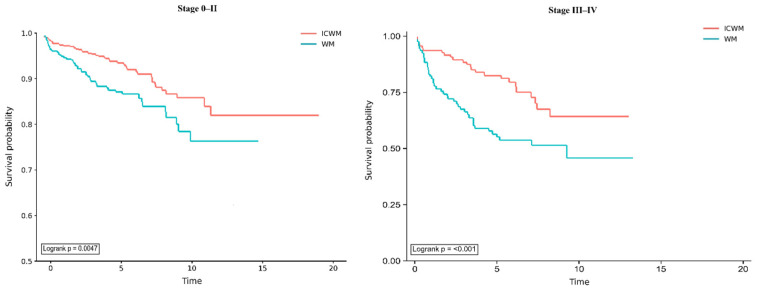
Kaplan–Meier survival curves by cancer stage for ICWM versus WM among BC survivors with depression.

Figure 3 presents Kaplan–Meier survival curves stratified by cancer stage for patients with post-breast cancer depression receiving ICWM versus WM. In the stage 0–II subgroup, survival was higher in the ICWM group than in the WM group (log-rank *p* = 0.0047). In the stage III–IV subgroup, the survival curves also differed between groups (*p* < 0.001). As expected, the Kaplan–Meier curves for advanced-stage disease (III–IV) showed more rapid declines than those for early-stage disease (0–II), consistent with poorer prognosis in patients with more advanced cancer. These findings suggest differences in survival between treatment groups within each stage stratum.

## 4. Discussion

### 4.1. Key Findings

This nationwide cohort study showed that ICWM was associated with lower all-cause mortality and lower healthcare expenditures than WM alone in breast cancer patients with comorbid depression within a universal health insurance system. In this population-based analysis of 1193 breast cancer patients with subsequent depression, ICWM users were younger and more often diagnosed at earlier stages than WM-only patients but also had a distinct comorbidity profile, indicating potential selection of more health-conscious or treatment-seeking individuals into ICWM. Despite longer fol-low-up and a higher number of total healthcare visits, ICWM had lower total, inpatient, and emergency expenditures per person-year, suggesting a shift toward lower acute-care expenditures and greater outpatient service use. Multivariable Cox models further demonstrated that ICWM was independently associated with lower all-cause mortality, with a stronger protective effect in patients with advanced-stage (III–IV) disease than in those with early-stage (0–II) disease.

### 4.2. Comparison with Previous Studies

Accumulating evidence from clinical and observational studies indicates that incorporating TCM as an adjunctive therapy can enhance immune responses, alleviate symptoms, and improve quality of life in patients with breast cancer [34,35,36,37,38]. Consequently, ICWM is attracting interest for its potential to improve outcomes while complementing mechanism-based Western interventions [35,36]. Our findings are consistent with a prior study indicating that adding traditional Chinese medicine (TCM) to standard oncologic treatment may improve survival and patient well-being in patients with breast cancer [15]. A longitudinal Taiwanese cohort further reported that, among long-term breast cancer survivors, CHM use was associated with a lower subsequent risk of depression among long-term BC survivors, supporting the real-world effectiveness of integrative care in this population [39]. In the present study, healthcare utilization outcomes, including frequent emergency department visits, inpatient admissions, and prolonged hospital stays, were selected as pragmatic indicators of clinical burden and healthcare resource use rather than direct measures of quality of life. This interpretation is supported indirectly by prior evidence showing that functional disability is associated with greater healthcare utilization among cancer survivors and that physical functional impairments in breast cancer patients and survivors may adversely affect activities of daily living, social and role functioning, and overall QoL [40,41]. Our findings extend this evidence by showing that among breast cancer patients who had already developed depression, ICWM was associated not only with improved survival but also with more favorable healthcare utilization patterns, particularly lower inpatient and emergency care expenditures. Nevertheless, because no validated QoL instruments were available in this study, these utilization measures should be interpreted as indirect proxy indicators rather than direct assessments of QoL.

Age and comorbidities also appeared to be important factors in prognosis. In early-stage disease, age ≥65 years and congestive heart failure were associated with higher all-cause mortality, underscoring the potential impact of aging and cardiovascular comorbidity even in patients with more favorable tumor stage, in line with prior work showing that breast cancer survivors with heart failure or cardiomyopathy face elevated risks of cardiovascular events, hospitalization, and mortality [42,43,44]. In advanced-stage disease, the associations of cardiometabolic comorbidities with all-cause mortality were not uniform across variables. Notably, diabetes was associated with a lower hazard ratio in the stage III–IV subgroup, whereas heart failure tended to be associated with worse prognosis, although some estimates did not reach statistical significance, possibly due to limited sample size and event numbers in these strata. Given the subgroup nature of this analysis and the possibility of residual confounding, these findings should be interpreted cautiously. This pattern is consistent with previous reports that pre-existing cardiometabolic conditions may adversely influence long-term outcomes among breast cancer patients [44,45]. Taken together, these results highlight the need for integrated cardio-oncology and metabolic management for BC patients with depression and multimorbidity and indicate that ICWM could be incorporated as part of a broader multidisciplinary strategy that addresses oncologic, cardiovascular, metabolic, and psychological needs simultaneously.

The stage-stratified Cox proportional hazards models and Kaplan–Meier analyses showed that ICWM was associated with lower all-cause mortality across cancer stages, with a larger effect estimate in stage III–IV disease than in stage 0–II disease. This finding is clinically relevant because patients with advanced BC and depression often experience a particularly high symptom burden, greater treatment toxicity, and more intense psychosocial stress, all of which have been linked to worse quality of life and poorer survival in previous studies of advanced breast and other cancers [7]. Prior integrative oncology and palliative care programs have also demonstrated that comprehensive symptom management, adjuvant therapy and psychosocial support can reduce hospitalizations, lessen opioid requirements, and improve fatigue, depression, and global quality of life among patients with advanced cancer, supporting the notion that integrative approaches may be especially beneficial in this setting [38,46]. Nevertheless, the mechanisms underlying this association cannot be determined from the present observational design. In addition to the potential therapeutic effects of TCM interventions, unmeasured factors such as patient beliefs, treatment expectations, psychological support, and other psychosocial influences may have contributed to the observed associations.

From a healthcare utilization and economic perspective, ICWM users demonstrated significantly longer follow-up duration alongside higher numbers of overall and outpatient visits per person-year, yet substantially fewer emergency visits and lower inpatient, emergency, and total healthcare expenditures per person-year compared with WM-only patients. This pattern reflects a fundamental redistribution of care from high expenditures, acute/reactive interventions toward sustained, lower-intensity ambulatory management. Such restructuring aligns with value-based oncology principles, where enhanced ambulatory monitoring—facilitated by frequent TCM clinic contacts—enables earlier detection and management of emerging symptoms, complications, or depressive exacerbations, thereby preventing costly downstream emergency department utilization and hospitalizations [47,48]. The longer follow-up observed in ICWM recipients, together with the sustained separation of the Kaplan–Meier curves without marked late divergence, suggests that the association observed in this study was not limited to a short-term phenomenon. This pattern may be indicative of better care continuity and lower acute-care utilization. This shift from reactive to proactive care delivery not only optimizes resource allocation but also embodies the core tenets of integrative oncology: achieving more favorable outcomes at lower or comparable cost through coordinated care pathways, improved treatment adherence, and holistic symptom palliation rather than mere volume reduction [48]. These findings underscore the potential clinical and economic value of ICWM as a complementary care model in breast cancer patients with comorbid depression. However, because this was a non-randomized observational study based on administrative data, and because the WM and ICWM groups differed in several baseline demographic and clinical characteristics, residual confounding and selection-related bias cannot be completely excluded. Therefore, these findings should be interpreted cautiously and not as definitive evidence of causality.

Nevertheless, the significantly lower emergency care expenditures observed among ICWM users suggest that integrating TCM into conventional cancer care may help reduce acute healthcare needs in breast cancer patients with comorbid depression. Along with the observed survival advantage, these findings highlight the potential clinical and economic value of ICWM in routine oncology care. The strengths of this study include the use of a nationwide claims database with near-complete population coverage, long-term follow-up (up to 17 years), and comprehensive capture of both WM and TCM services within a unified health system. Although the use of a nationwide claims database with near-complete population coverage strengthens the representativeness of the study population within Taiwan, it does not in itself guarantee the generalizability of these findings to other healthcare systems, cultural settings, or patient populations. Future prospective and mechanism-oriented studies across different healthcare contexts are needed to further clarify the pathways through which ICWM may influence survival and acute care utilization and to determine the broader applicability of these findings.

### 4.3. Limitations

Several limitations of this study should be acknowledged. First, baseline characteristics differed between the groups as patients in the ICWM group tended to be younger and had fewer comorbidities. Although these variables were adjusted for in the multivariable regression models, residual confounding and selection bias cannot be completely excluded. Second, some expenditure-related variables were likely right-skewed; therefore, although the large sample size supports the relative robustness of the independent *t*-test for comparing arithmetic mean values, these results should still be interpreted with caution. Third, because no validated quality-of-life (QoL) instrument was available in the claims database, healthcare utilization outcomes should be regarded as proxy indicators rather than direct measures of patient-reported QoL. Fourth, because ICWM exposure was defined according to the accumulation of at least 30 cumulative days of Chinese herbal medicine use during follow-up, immortal time bias could not be completely excluded. The observed association between ICWM use and survival outcomes should accordingly be interpreted with caution. Future studies using larger datasets, propensity score-based approaches, nonparametric or other robust statistical methods, and validated patient-reported outcome measures are warranted to further verify these findings.

## 5. Conclusions

This nationwide cohort study showed that ICWM was associated with better survival, lower healthcare expenditures, and more favorable healthcare utilization patterns than WM alone among breast cancer patients with comorbid depression. ICWM users, who were younger and more often diagnosed at earlier stages despite distinct comorbidity profiles, had longer follow-up and greater outpatient service use, along with lower per-person-year expenditures for inpatient, emergency, and total care. However, because this was a retrospective observational study, causal inference is limited, and the observed associations should be interpreted cautiously.

## Figures and Tables

**Table 1:** Baseline characteristics of patients with post-breast cancer depression.

		Post-Breast Cancer Depression (n = 1193)		*p*-Value
		WM (n = 488)	ICWM (n = 705)	
		N (%)	N (%)	
Follow-up time (mean ± std)		5.03 ± 3.37	5.52 ± 3.47	0.471
Age group	<50	254 (52.05)	448 (63.55)	0.001
	50–64	135 (27.66)	170 (24.11)	
	≥65	99 (20.29)	87 (12.34)	
Age (mean ± std)		54.95 ± 11.56	51.31 ±10.30	0.001
Height (mean ± std)		1.56 ± 0.06	1.56 ±0.05	0.149
Weight (mean ± std)		60.18 ± 9.98	58.45 ± 8.74	0.047
BMI (mean ± std)		24.68 ± 4.55	23.76 ± 4.17	0.001
CCI scores	0–1	255 (52.25)	347 (49.22)	0.426
	2–3	152 (31.15)	252 (35.74)	
	4–5	56 (11.48)	74 (10.5)	
	6+	25 (5.12)	32 (4.54)	
CCI scores (mean ± std)		1.85 ± 1.97	1.89 ± 1.98	0.905
Treatment modality	OP only	57 (11.68)	84 (11.91)	0.006
	OP + RT	17 (3.48)	38 (5.39)	
	OP + HT	92 (18.85)	92 (13.05)	
	OP + CT	54 (11.07)	55 (7.8)	
	Others	268 (54.92)	436 (61.84)	
Stage group	0–II	372 (76.23)	564 (80.00)	0.021
	III–IV	97 (19.88)	101 (14.33)	
	unknown	19 (3.89)	40 (5.67)	
Insured category	Employer /professional	11 (2.59)	20 (2.72)	0.154
	General	384 (90.57)	683 (93.05)	
	Parental leave /Dependent	29 (6.84)	31 (4.22)	
Taxanes		144 (29.51)	211 (29.93)	0.876
Underlying disease				
HTN		56 (11.48)	40 (5.67)	<0.001
HLD		178 (36.48)	214 (30.35)	0.027
CHF		31 (6.35)	24 (3.40)	0.017
DM		124 (25.41)	108 (15.32)	0.001
Gout		83 (17.01)	83 (11.77)	0.010
Pneumonia		166 (34.02)	288 (40.85)	0.017
Major bleeding		106 (21.72)	198 (28.09)	0.013
GI bleeding		115 (23.57)	212 (30.07)	0.013
Anemia		71 (14.55)	143 (20.28)	0.011
IHD		98 (20.08)	107 (15.18)	0.027

Abbreviation: WM: Western medicine; ICWM: Integrated Chinese and Western medicine; mean ± std: mean ± standard deviation; CCI: Charlson Comorbidity Index; BMI: Body mass index; OP: Operation; RT: Radiotherapy; HT: Hormone Therapy; CT: Chemotherapy; HTN: Hypertension; HLD: Hyperlipidemia; CHF: congestive heart failure; DM: diabetes mellitus; GI bleeding: Gastrointestinal bleeding; IHD: Ischemic heart disease.

**Table 2:** Summary of healthcare expenditures between the ICWM and WM groups.

		Post-Breast Cancer Depression (n = 1193)	
		WM (n = 488)	ICWM (n = 705)
Overall expenditure			
Follow-up time (years), mean ± sd		9.99 ± 3.16	10.61 ± 2.46
Total expenditure, sum		507,471,621	623,485,606
Expenditure per person-year	mean ± std	162,084 ± 268,369	114,145 ± 189,454
	Median [IQR]	50,513 [24, 950–117, 302]	48,208 [25, 774–90, 174]
Inpatient care			
Follow-up time (years), mean ± std		9.88 ± 3.39	10.46 ± 2.60
Total expenditure, sum		195,542,868	203,072,618
Expenditure per person-year	mean ± std	96,916 ± 208,929	58,381 ± 125,912
	Median [IQR]	10,347 [0–40, 674]	7165 [0–24, 640]
Outpatient care			
Follow-up time (years), mean ± std		8.23 ± 3.67	9.44 ± 3.12
Total expenditure, sum		311,928,753	420,412,988
Expenditure per person-year	mean ± std	89,397 ± 141,724	74,566 ± 123,256
	Median [IQR]	39,884 [24, 132–69, 612]	41,345 [26, 850–67, 107]
Emergency care			
Follow-up time (years), mean ± std		10.01 ± 3.18	10.73 ± 2.56
Total expenditure, sum		7,239,801	6,847,850
Expenditure per person-year	mean ± std	2752 ± 5790	1588 ± 2688
	Median [IQR]	467.4 [0–1937]	296.7 [0–1163]

Abbreviation: WM: Western medicine; ICWM: Integrated Chinese and Western medicine; mean ± std: mean ± standard deviation; IQR: Interquartile Range.

**Table 3:** Summary of healthcare visit counts in the WM and ICWM groups.

		Post-Breast Cancer Depression (n = 1193)	
		WM (n = 488)	ICWM (n = 705)
Overall Visits			
Total number of visits		142,382	253,291
Number of visits per person-year	mean ± std	34.14 ± 18.78	39.63 ± 20.00
	Median [IQR]	30.1 [21.8–41.7]	35.3 [25.3–50.3]
Inpatient Visit			
Total number of visits		4182	5614
Number of visits per person-year	mean ± std	1.94 ± 4.41	1.44 ± 2.69
	Median [IQR]	0.6 [0.3–1.5]	0.5 [0.2–1.4]
Outpatient Visit			
Total number of visits		138,200	247,677
Number of visits per person-year	mean ± std	32.64 ± 17.24	38.62 ± 19.34
	Median [IQR]	29.2 [21.2–39.8]	34.3 [24.7–48.1]
Emergency Visit			
Total number of visits		1731	1930
Number of visits per person-year	mean ± std	0.63 ± 1.09	0.43 ± 0.50
	Median [IQR]	0.4 [0.2–0.6]	0.3 [0.1–0.5]

Abbreviation: WM: Western medicine; ICWM: Integrated Chinese and Western medicine; mean ± std: mean ± standard deviation; IQR: Interquartile Range.

**Table 4:** Multivariable Cox proportional hazards analysis among different stages.

		Stage 0–II		Stage III–IV	
		aHR (95% C.I)	*p*-Value	aHR (95% C.I)	*p*-Value
Group	WM (Ref.)	1		1	
	ICWM	0.61 (0.39–0.94)	0.0242	0.38 (0.21–0.67)	<0.001
Age group	<55 (Ref.)	1		1	
	55–64	1.32 (0.75–2.30)	0.3335	1.38 (0.74–2.60)	0.3155
	≥65	3.10 (1.72–5.59)	<0.001	2.04 (0.90–4.64)	0.0887
BMI	Underweight (Ref.)	1		1	
	Normal	0.86 (0.30–2.46)	0.7837	1.31 (0.32–5.28)	0.7079
	Overweight	1.00 (0.37–2.75)	0.9971	1.34 (0.36–4.94)	0.6602
	Unknown	0.94 (0.37–2.40)	0.8928	1.63 (0.47–5.59)	0.4395
Treatment modality	OP only (Ref.)	1		1	
	OP + RT	0.39 (0.09–1.71)	0.2131	0.24 (0.03–2.28)	0.2151
	OP + HT	0.64 (0.33–1.25)	0.1956	0.62 (0.15–2.55)	0.5118
	OP + CT	0.57 (0.25–1.28)	0.1739	1.45 (0.39–5.40)	0.5825
	Others	0.58 (0.33–1.02)	0.0603	0.56 (0.19–1.69)	0.3054
HTN		1.33 (0.71–2.51)	0.3769	1.22 (0.47–3.20)	0.6851
HLD		0.93 (0.57–1.53)	0.7781	1.21 (0.64–2.30)	0.5518
CHF		2.95 (1.53–5.72)	0.0013	2.77 (0.91–8.41)	0.0716
DM		0.84 (0.49–1.44)	0.5360	0.29 (0.13–0.68)	0.0042
Gout		1.10 (0.62–1.96)	0.7355	1.31 (0.66–2.63)	0.4437
Pneumonia		1.24 (0.81–1.91)	0.3207	0.76 (0.43–1.37)	0.3671
Major bleeding		1.12 (0.65–1.91)	0.0937	1.63 (0.86–3.09)	0.1367
GI bleeding		1.26 (0.70–2.26)	0.6851	1.19 (0.57–2.50)	0.6377
Anemia		1.44 (0.87–2.43)	0.1686	0.99 (0.50–1.95)	0.9792
IHD		0.58 (0.32–1.03)	0.0606	0.64 (0.30–1.38)	0.2547

Abbreviation: aHR: adjusted hazard ratio; WM: Western medicine; ICWM: Integrated Chinese and Western medicine; BMI: Body mass index; OP: Operation; RT: Radiotherapy; HT: Hormone Therapy; CT: Chemotherapy; HTN: Hypertension; HLD: Hyperlipidemia; CHF: congestive heart failure; DM: diabetes mellitus; GI bleeding: Gastrointestinal bleeding; IHD: Ischemic heart disease.

## Data Availability

The datasets analyzed in this study were obtained from Taiwan’s National Health Insurance Research Database (NHIRD) and Taiwan Cancer Registry (TCR), which are managed by the Taiwan Ministry of Health and Welfare (MOHW). Due to data privacy regulations, these de-identified datasets are provided to researchers through a secure, controlled-access process and are not publicly available. Researchers interested in accessing these datasets for replication or further analysis may submit formal applications through the Health and Welfare Data Science Center application portal: https://dep.mohw.gov.tw/DOS/mp-113.html. (accessed on 8 August 2024).

## Abbreviations

The following abbreviations are used in this manuscript:

WM	Western medicine
ICWM	Integrated Chinese and Western medicine
CCI	Charlson Comorbidity Index
BMI	Body mass index
OP	Operation
RT	Radiotherapy
HT	Hormone Therapy
CT	Chemotherapy
HTN	Hypertension
HLD	Hyperlipidemia
GI bleeding	Gastrointestinal
IHD	Ischemic heart disease

## Appendix A. Code for Comorbidities

	**ICD-9-CM**	**ICD-10-CM**
**CCI score**		
Myocardial infarct	410	I21–22
Congestive heart failure	428.0, 428.1, 428.9	I50
Cerebrovascular disease	430–438	I60–I63, I65–I69, G45–G46
Connective tissue disease	710, 714, 725	M05, M06, M32, M33.20, M33.29, M34, M35.3
Peripheral vascular disease	250.7, 440.2–3, 440.8–9, 443, 444.22, 444.8, 447.8–9	E08.51–52, E08.59, E09.51–52, E09.59, E10.51–52, E10.59, E11.51–52, E11.59, E13.51–52, E13.59, I70.2–I70.9, I73, I74.2–I74.9, I75.011–I75.029, I75.89, I77.3, I77.89, I77.9, I79.1, I79.8
Dementia	290	F03.90, F05, F01.50, F01.51
COPD	490–492, 496	J40–J44, J47
Ulcer disease	531.XX, 532.XX, 533.XX, 534.XX	K25.X, K26.X, K27.X, K28.X, K31.82, K56.60
Mild liver disease	571.2, 571.4X, 571.5, 571.6	K70.2, K70.3X, K73.X, K74.X, K74.60, K74.69, K75.4
Hemiplegia	342	G81
Moderate-to-severe renal disease	582.0, 582.1, 582.2, 582.4, 582.81, 582.89, 582.9, 583.0, 583.1, 583.2, 583.4, 583.6, 583.7, 583.9, 588.1, 588.8, 588.9, 583.81, 583.89, 585, 586, 588	E10.21, E11.21, N03.0, N03.1, N03.2, N03.3, N03.4, N03.5 N03.6, N03.7, N03.8, N03.9, N05.0, N05.1, N05.2, N05.3, N05.4, N05.5, N05.6, N05.7, N05.8, N05.9, N06.0, N06.1, N06.2, N06.3, N06.4, N06.5, N06.6, N06.7, N06.8, N06.9, N07.0, N07.1, N07.2, N07.3, N07.4, N07.5, N07.6, N07.7, N07.8, N07.9, N08, N14.0, N14.1, N14.2, N14.3, N14.4, N15.0, N15.8, N15.9, N16, N17.1, N17.2, N18.4, N18.5, N18.6, N18.9, N19, N25.0, N25.1, N25.81, N25.89, N25.9

## Appendix B. Subgroup Analysis of WM and ICWM Treatments and Mortality Risk in BC Patients with Depression

**Subgroup**		**aHR (95% C.I.)**	***p*-Value**
Age group	<55	0.52 (0.33–0.82)	0.004
	55–64	0.52 (0.29–0.92)	0.024
	≥65	0.65 (0.37–1.16)	0.145
BMI	Underweight	0.92 (0.34–2.55)	0.878
	Normal	0.69 (0.30–1.59)	0.380
	Overweight	0.75 (0.39–1.45)	0.392
CCI	0	0.44 (0.25–0.78)	0.005
	1	0.42 (0.20–0.87)	0.019
	2	0.56 (0.28–1.12)	0.100
	3	0.74 (0.34–1.63)	0.458
	4	1.28 (0.37–4.38)	0.697
	5	0.58 (1.34–2.47)	0.461
	6+	0.24 (0.08–0.70)	0.009
Treatment modality	OP only	0.77 (0.34–1.71)	0.515
	OP + RT	0.2 (0.02–2.19)	0.187
	OP + HT	0.33 (0.14–0.80)	0.014
	OP + CT	0.45 (0.17–1.21)	0.113
	Others	0.52 (0.35–0.77)	<0.001
Stage	I	0.62 (0.27–1.43)	0.259
	II	0.56 (0.35–0.91)	0.020
	III	0.45 (0.26–0.80)	0.006
	IV	0.43 (0.15–1.26)	0.124
Insured category	Employer/professional	0.19 (0.02–2.15)	0.180
	General	0.51 (0.37–0.69)	<0.001
	Parental leave/Dependent	0.66 (0.19–2.28)	0.511
Taxanes	Yes	0.62 (0.42–0.90)	0.011
